# Complete Genome Sequence of an Efficient Vitamin D_3_-Hydroxylating Bacterium, Pseudonocardia autotrophica NBRC 12743

**DOI:** 10.1128/MRA.01105-18

**Published:** 2018-09-27

**Authors:** Keitaro Yoshida, Yoshiaki Yasutake, Tomohiro Tamura

**Affiliations:** a Bioproduction Research Institute, National Institute of Advanced Industrial Science and Technology (AIST), Sapporo, Japan; b Laboratory of Molecular Environmental Microbiology, Graduate School of Agriculture, Hokkaido University, Sapporo, Japan; c Computational Bio Big-Data Open Innovation Laboratory (CBBD-OIL), AIST, Sapporo, Japan; Georgia Institute of Technology

## Abstract

Pseudonocardia autotrophica NBRC 12743 contains a cytochrome P450 vitamin D_3_ hydroxylase, and it is used as a biocatalyst for the commercial production of hydroxyvitamin D_3_, a valuable compound for medication. Here, we report the complete genome sequence of P. autotrophica NBRC 12743, which could be useful for improving the productivity of hydroxyvitamin D_3_.

## ANNOUNCEMENT

Hydroxylated vitamin D_3_ (VD_3_) and its derivatives are useful as pharmaceuticals for treating VD_3_ deficiency-related diseases in clinical settings. Several actinomycete species capable of hydroxylating VD_3_ have been isolated to date (1–4). Among these isolated bacteria, Pseudonocardia autotrophica NBRC 12743 showed the highest VD_3_-hydroxylating activity, and the strain has thus been applied to one-pot commercial production of calcitriol (1α,25-dihydroxyvitamin D_3_) (1, 5). The gene encoding VD_3_ hydroxylase (Vdh) was successfully cloned and sequenced (GenBank accession number AB456955) (2), showing that Vdh is a cytochrome P450 monooxygenase. Directed-evolution studies together with three-dimensional structure analyses of Vdh have also been performed to improve its enzymatic activity (2, 6–8). A draft genome sequence of this strain with 117 contigs was reported recently (9).

Here, we sequenced and assembled the genome of P. autotrophica strain NBRC 12743. The P. autotrophica strain was cultured in Luria-Bertani broth for 72 h at 28°C, and genomic DNA was isolated by the conventional phenol-chloroform extraction method. The genome was sequenced using PacBio RS II and Illumina HiSeq 2500 sequencers with 100-bp paired-end systems (Hokkaido System Science Co., Ltd., Sapporo, Japan). The PacBio raw reads were filtered using the SMRT Analysis program (version 2.3.0), and 115,874 subreads (886,044,835 bp) were obtained. These subreads were assembled with the Hierarchical Genome Assembly Process (HGAP) (version 2) (10), resulting in a circular chromosomal sequence with a mean coverage of 67-fold. The HiSeq paired-end reads were filtered with Trimmomatic (version 0.38) to remove low-quality reads with the parameters SLIDINGWINDOW:20:20 and MINLEN:50 (11), and 44,241,534 reads (4,438,360,972 bp) were obtained. These filtered reads were then mapped to the chromosome sequence using the BWA-MEM algorithm (version 0.7.12) with a seed length of 19, and sequencing errors were corrected using the Genome Analysis Toolkit (GATK) pipeline (version 4.0.6.0) with default parameters (12, 13). The plasmid sequence was assembled from the hybrid reads (both the filtered PacBio and HiSeq reads), using the Unicycler hybrid assembler (version 0.4.6) with default parameters (14). Finally, we identified another circular sequence which does not overlap the chromosome sequence. Genes were annotated using the DDBJ Fast Annotation and Submission Tool (DFAST) pipeline (15).

The genome of NBRC 12743 was inferred to contain two circular sequences with a length of 7,246,130 bp with 72.9% G+C content and a length of 289,155 bp with 70.2% G+C content. The genome contains 7,132 putative coding sequences, 12 rRNAs (4 23S, 16S, and 5S rRNAs each), and 70 tRNAs. We searched for genes encoding cytochrome P450s using the Microbial Genome Annotation Pipeline (http://www.migap.org/) and found that NBRC 12743 possesses 28 cytochrome P450 genes, including the gene encoding Vdh. We found that all of the cytochrome P450 genes exist in the chromosome. The genomic information reported in this study will be helpful for engineering this bacterium as a recombinant expression host of P450 and for improving expression levels of Vdh in order to achieve more efficient bioproduction of hydroxyvitamin D_3_.

### Data availability.

The genome sequence has been deposited in DDBJ/ENA/GenBank under the accession numbers AP018920 and AP018921. The raw sequencing reads have been deposited in the DDBJ Sequence Read Archive under the accession numbers DRX139961 and DRX139962.
